# First isolation of bovine coronavirus with a three-amino-acid deletion in the N gene causing severe respiratory and digestive disease in calve

**DOI:** 10.3389/fmicb.2024.1466096

**Published:** 2024-10-01

**Authors:** Siyuan Li, Xuesong Yuan, Li Mao, Xuhang Cai, Xingang Xu, Jizong Li, Bin Li

**Affiliations:** ^1^Key Laboratory of Veterinary Biological Engineering and Technology Ministry of Agriculture, Jiangsu Key Laboratory for Food Quality and Safety-State Key Laboratory Cultivation Base of Ministry of Science and Technology, Institute of Veterinary Medicine, Jiangsu Academy of Agricultural Sciences, Nanjing, China; ^2^College of Veterinary Medicine, Northwest A&F University, Yangling, China; ^3^College of Veterinary Medicine, Nanjing Agricultural University, Nanjing, China; ^4^School of Food and Biological Engineering, Institute of Life Sciences, Jiangsu University, Zhenjiang, China; ^5^Jiangsu Co-Innovation Center for the Prevention and Control of Important Animal Infectious Disease and Zoonose, Yangzhou University, Yangzhou, PR China

**Keywords:** bovine coronavirus, pathogenicity, N gene, mutation, GIIb genotype

## Abstract

Bovine coronavirus (BCoV), a persistent threat to global cattle industry, has caused significant economic losses worldwide. In this study, a viral strain was isolated from the intestinal content of a diseased calve, and identified by cytopathic effects observation, indirect immunofluorescence assay and electron microscopy. Results showed that BCoV NXWZ2310 belonging to the GIIb genotype and has a three-amino-acid deletion in the serine-rich region of the N gene. Importantly, the BCoV NXWZ2310 strain exhibited strong pathogenicity, causing nasal discharge and watery diarrhea in calves for 8 and 10 days, respectively. Viral shedding was detected in nasal, throat and rectal swabs at levels reaching 10^6.228^ copies/mL, 10^5.0^ copies /mL and 10^6.692^ copies/mL, respectively. Pathological examination showed that NXWZ2310 resulted in parenchymal lesions of the pulmonary lobe and significant intestinal lesions. Both the lungs and intestines displayed marked microscopic lesions with clear viral antigens present. BCoV NXWZ2310 strain with N-gene deletion mutations, caused severe respiratory and digestive disease in calves. Therefore, effective strategies are needed for the prevention and control of this isolate.

## 1 Introduction

Bovine coronavirus (BCoV), an enveloped, single-stranded, positive-sense RNA virus, belongs to the genus *Betacoronavirus* (lineage A) in the family *Coronaviridae* and order *Nidovirales*. This genus Betacoronavirus is also important for humans as it includes severe acute respiratory syndrome-related coronavirus (SARS-CoV), Middle-East respiratory syndrome-related coronavirus, and SARS-CoV-2 (Masters, 2006; Zaki et al., 2012). Other members of this genus include human OC43 coronavirus (HCoV-OC43), mouse hepatitis virus (MHV), equine coronavirus (ECoV), porcine hemagglutinating encephalomyelitis virus (PHEV) and canine respiratory coronavirus (CRCoV). BCoV displays significant diversity and can be primarily divided into GI and GII groups. Upon further classification, it was found that classical strains detected in different regions cluster to form the GIa subtype. Most BCoV strains from Europe belong to the GIb subtype, the majority of BCoV strains from Asia and America cluster into the GIIb group, with the exception of some strains detected in South Korea, which belong to the GIIa subtype. The genome of BCoV is approximately 31 kb. Two-thirds of it encode the polyprotein (pp) 1a and pp1ab, while the remaining genome contains ORFs that encode five structural proteins (spike [S], envelope [E], membrane [M], nucleocapsid [N], and hemagglutinin-esterase [HE]), and the accessory proteins (ns2, ns4.9, ns4.8, ns12.7). BCoV is widespread globally and causes clinical symptoms such as neonatal calf diarrhea, winter dysentery, and decreased milk production and is increasingly reported in respiratory infections (Rahe et al., 2022; Socha et al., 2022). BCoV is considered one of the earliest coronaviruses to cross species barriers and can infect cows, giraffes (Hasoksuz et al., 2007), sheep (Smith et al., 2022), and other ruminants (Alekseev et al., 2008). It has also recently reported in rodents (Xu et al., 2023). BCoV exhibits the tissue tropism for both the intestine and respiratory tract, and can cause serious damage to both organs. In 2020, a total of 1383 samples were collected from cattle exhibiting diarrhea symptoms, BCoV was detected in respiratory samples with a positive rate of 21.53%, and with 12.20% in nasal swab samples in China (Zhu et al., 2022a). Thus, this virus has attracted increasing attention due to its broad host range and the extensive economic losses it causes.

Like other coronaviruses, BCoV can quickly adapt to changing ecological niches due to its high mutation rates and recombination frequencies. For example, many studies have reported the emergence of mutant strains, which include deletions and insertions of 4 amino-acid in the HE protein and deletions in nsp4.8 and nsp4.9 (He et al., 2019; Workman et al., 2022). HE mutants can cause changes in sugar tropism and increase the host range of BCoV by expanding the number and types of cells the virus can infect (Langereis et al., 2012). Truncation of nsp4.8 and nsp4.9 has been reported to be associated with host adaptability in HCoV-OC43, (Vijgen et al., 2005). Whether this truncation is related to changes in the tissue tropism and host selectivity of BCoV remains uncertain, and mutations in other Betacoronaviruses should also be investigated.

The primary function of the coronavirus N protein is to wrap the viral genome into long, flexible, helical ribonucleoprotein complexes, protecting the genome and ensuring its timely replication and reliable transmission (McBride et al., 2014). A recent study revealed that during SARS-CoV-2 ribonucleosomal assembly, amino-acid deletion of the N protein was involved in compacting the viral RNA genome into ribonucleoprotein complexes (Adly et al., 2023). Additionally, interaction between the BCoV N protein and the genome ends, along with amino-acid deletion in the N protein, is involved in genome circularization and negative-strand RNA synthesis (Lo et al., 2019). Three-amino-acid deletions in the N gene of BCoV had been detected in Japan, France, the United States and China. In addition, twelve genome sequences have been uploaded to NCBI. However, no strain has been successfully isolated, and the pathogenicity of the strain is unknown.

Here, we first isolated BCoV strain NXWZ2310, genotype GIIb, with N-gene deletion from calves with severe diarrhea in Wuzhong City, Ningxia Province, China. We investigated its pathogenicity in 7-day-old calves. BCoV NXWZ2310 strain caused nasal discharge and watery diarrhea and resulted in high levels of viral shedding and gross pathological and histological changes in the calves’ intestines and lungs. Our findings will enable better understanding the pathogenicity of emerging BCoV

## 2 Materials and methods

### 2.1 Sample collection and processing

Fecal samples were collected from cattle with severe diarrhea from Wuzhong City, NingXia Province, China, on 14 October 2023. All samples were immediately transferred to 2-mL tubes and completely mixed with 800 μL of sterile phosphate-buffered saline (PBS), then centrifuged at 12,000 r/min at 4°C for 5 min. The supernatant was collected and filtered through 0.22-μm column filters to remove bacteria and contaminants.

RNA was extracted using the FastPure Cell/Tissue Total RNA Isolation Kit V2 (Vazyme, China) according to the manufacturer’s instructions. The RNA was then mixed with HiScript II Q RT SuperMix (Vazyme, China) for reverse transcription into cDNA. RT-PCR was performed to detect BCoV, bovine rotavirus (BoRV), bovine viral diarrhea virus (BVDV) and bovine astrovirus (BoAstV) using Green Taq Mix (Vazyme, China). Amplification products were detected by electrophoresis in 1.2% agarose gels.

### 2.2 Viral isolation and identification

BCoV-positive samples were filtered through a 0.22-μm filter. The supernatant was inoculated onto Human Rectal Tumor 18 cells (HRT-18) in 24-well plates. The cells were placed in Dulbecco’s modified Eagle’s medium (DMEM; Sigma, CA, USA) containing 30 μg/mL trypsin and adsorbed for 3 h at 37°C with 5% CO2. The cells were then incubated at 37°C with 5% CO2 for 3 days to observe the cytopathic effect (CPE), then serially passaged for 10 generations in HRT-18 cells. The harvested viruses were identified via immunofluorescence assay (IFA).

### 2.3 Passage of BCoV in HRT-18 cells

HRT-18 cells grown to a monolayer were treated with DMEM containing 30 μg/mL trypsin and viral supernatant, then incubated at 37°C with 5% CO_2_ for 3 h. The cells were maintained in DMEM with 10 μg/mL trypsin for 3 days. When the cells exhibited 70% CPE, they were subjected to two freeze-thaw cycles. The viral stock was collected as passage (P)0 and serially passaged for 10 generations.

### 2.4 Characterization by electron microscopy

The viruses were harvested from infected HRT-18 cells. The supernatant was centrifuged at 12,000 r/min for 30 min, then ultracentrifugation at 100,000 g for 3 h in a Beckman Optima MAX-XP (Beckman, USA) and resuspended with PBS. Viral samples (10 μL) were added to a carbon-coated grid for 1 min and stained with 2% phosphotungstic acid for 1 min. The viral morphology was observed using a Tecnai G2 Spirit TWIN transmission electron microscope (Philips, Tecnai 12, Netherlands).

### 2.5 IFA

HRT-18 cells were infected with BCoV at a multiplicity of infection (MOI) of 0.1. The cells were then fixed in 4% paraformaldehyde for 15 minutes and blocked with 5% skimmed milk powder for 1 h. The treated cells were incubated with anti-BCoV-S1 polyclonal antibody (prepared by our lab; 1:1,000) at 37°C for 1 h, then incubated with Cy3-conjugated goat anti-mouse IgG H&L (Beyotime, Shanghai, China) for 1 h at 37°C. The cells were then washed with PBS, stained with 4′,6-diamidino-2-phenylindole (DAPI; Beyotime, Shanghai, China) for 15 min, and finally analyzed under a fluorescence microscope (Nikon, Japan).

### 2.6 Detection of exogenous viruses

To verify whether the isolated strain was contaminated with other viruses, primers were designed using Primer Premier 5 to detect BoRV, BoAstV, enterovirus, adenovirus coronavirus and BVDV (Table 1). Viral genomic RNA was extracted from the harvested supernatant and detected by RT-PCR as description in section 2.1.

**TABLE 1:** Sequences of primers used in this study.

Primer name	Sequence (5′-3′)	Production/ bp
BCoV-F	5′-AAGGTGTGCCTATTGCACCAG-3′	500
BCoV-R	5′-GCTTAGTTACTTGCTGTGGC-3′	
RV-vp6-F	5′-ACCACCAAATATGACACCAGC-3′	294
RV-vp6-R	5′-CATGCTTCTAATGGAAGCCAC-3′	
BVDV-F	5′-AGCGGGGATAAGGTTGGAAA-3′	200
BVDV-R	5′-ACCTGCAGCCCCTTTTCTAT-3′	
BoAstV-F	5′-GAYTGGACBCGHTWTGATGG-3′	418
BoAstV-R	5′-KYTTRACCCACATNCCAA-3′	
EV-F	5′- CTCCACTACGGTGCACCAGT -3′	250
EV-R	5′- CCCATCACTCAGAGCTACCAC -3′	
AdV-F	5′- TCAGGACCGCCTGGATCATA -3′	796
AdV-R	5′- TCAGCCACGCAAAGCCATTT -3′	
qBCoV-F	5′-AAGGTGTGCCTATTGCACCAG-3′	101
qBCoV-R	5′-GCTTAGTTACTTGCTGTGGC-3′	
BCoV-N-probe	FAM-CTATCTTGGAACAGGACCGCATGCCA-BHQ1	

### 2.7 Sequence analysis

The whole genome sequence of the NXWZ2310 isolates was obtained via next-generation sequencing by Shanghai Tanpu Biotechnology Co., Ltd (Shanghai, China). The sequences were aligned using ClustalW in MEGA11 software, and the phylogenetic tree was constructed using the maximum likelihood method. Homology between NXWZ2310 and the reference sequences was analyzed using Megalign in DNAstar 7.0.

### 2.8 Animal experimental design

The Jiangsu Academy of Agricultural Sciences Institutional Animal Care and Use Committee approved all animal experimental protocols (IACUC-2023-11-001). The neonatal calves were tested negative for BCoV, BoRV, BoAstV, enterovirus, adenovirus, coronavirus and BVDV by RT-PCR. No neutralizing antibodies against BCoV were detected in the sera of calves using serum neutralization assay. Twelve calves were randomly divided into four groups. Each group consisting of three calves was housed in a separate pen for clinical examination and fed milk powder every 6 h. At 7 days of age, each calf was orally inoculated with BCoV NXWZ2310 strain (intranasally: 5 mL; orally: 5 mL, 10^5.0^ TCID_50_/mL, P5). The mock group was inoculated with equal volume of DMEM containing 10 μg/mL trypsin. Clinical symptoms, including diarrhea and respiratory signs, were observed twice daily, and nasal, throat, and rectal swabs were collected daily for qRT-PCR. Calves were euthanized at 7 and 14 days post-infection (DPI), and tissues were collected for viral load analysis via qRT-PCR and fixed in 4% paraformaldehyde solution for histopathological analysis.

### 2.9 qRT-PCR

Total RNA was extracted from swabs, tissues and culture samples, then reverse transcribed into cDNA as description in section 2.1. BCoV-N gene transcripts were quantified via qRT-PCR with the probes and primers listed in Table 1. The qRT-PCR assays were performed on an ABI QuantStudio 6 (Applied Biosystems, CA, USA), and each samples were amplified at least three times.

### 2.10 Histopathology and IFA analysis

Tissue samples from various organs, including the heart, liver, spleen, lungs, kidneys, lung lymph nodes, mesenteric lymph nodes, and intestinal sections, were collected during necropsy. The samples were fixed in 4% paraformaldehyde for 48 h, routinely processed, and embedded in paraffin. Sections of the embedded tissues were then cut and stained with routine hematoxylin and eosin. Slides were examined under conventional light microscopy.

BCoV-specific antigens were detected on selected formalin-fixed and paraffin-embedded sections using anti-BCoV-S1 polyclonal antibody with FITC-conjugated goat anti-mouse IgG secondary antibodies (Beyotime, Shanghai, China). The nuclei were stained using a 1:5000 dilution of DAPI (Beyotime, Shanghai, China) for 10 min. The fluorescent images were observed with a fluorescence microscope.

### 2.11 Statistical analysis

Statistical significance was determined using t-tests, multiple-comparison t-tests and one-way analysis of variance with Tukey’s multiple-comparison tests using GraphPad Prism 5 software (GraphPad Software, Inc., San Diego, CA, USA). Lines indicate direct comparisons between groups. Asterisks denote statistical significance, where *P < 0.05, **P < 0.01, and ***P < 0.001.

## 3 Results

### 3.1 Viral isolation, viral growth characterization and morphological observation

**FIGURE 1:**
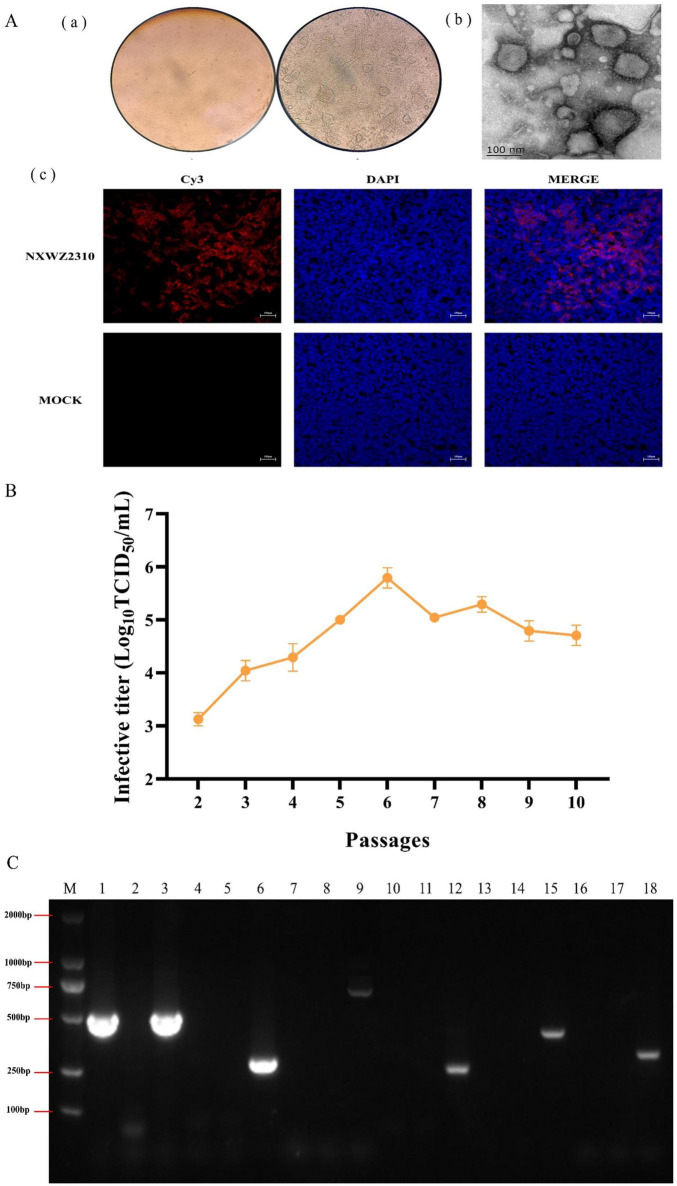
Biological characterization of BCoV NXWZ2310 strain. **(A)** (a) A CPE was observed in HRT-18 cells. 1. Control cells; 2. Challenge cells; Scale bar: 400 μm; (b) Electron microscope was used to observe BCoV NXWZ2310 strain; Scale bar: 100 nm; (c) IFA was used to detect BCoV NXWZ2310 strain; Scale bar: 200 μm; **(B)** Virus titers in HRT-18 cells over 10 serial passages. **(C)** Exogenous virus detection. M: DL2000 maker; 1,4,7,10,13,16: NXWZ2310 strain; 2,5,8,11,14,17: Negative control; 3,6,9,12,15,18: positive plasmid of BCoV, EV, BoAdV, BVDV, BoAstV, and BoRV, respectively.

BCoV NXWZ2310 was isolated from fecal samples of severe diarrhea. No pathogens other than BCoV were detected in these samples. When passaged to three generations, obvious CPEs, such as enlarged and rounded cells and debris, were observed microscopically (Figure 1A).

BCoV NXWZ2310 strain was confirmed via IFA using S1-specific polyclonal antibodies. Most cells showed specific red fluorescence, and the cytoplasm contained S1 protein. Conversely, the mock samples showed no specific red fluorescence [(Figure 1A(c)].

BCoV NXWZ2310 strain were serially passaged in HRT-18 cells for 10 passages. Initial titers were high at 10^3.0 ± 0.125^ TCID_50_/ml at P2, and yielded up to > 10^6.0 ± 0.191^ TCID_50_/ml by P6 (Figure 1B).

The transmission electron microscopy images showed virions of approximately 100 nm in diameter, displaying similar morphology to coronaviruses similar to BCoV [Figure 1A(b)]. RT-PCR showed that the BCoV NXWZ2310 strain were positive only for BCoV and negative for BoRV, BoAstV, enterovirus, adenovirus and BVDV (Figure 1C).

### 3.2 BCoV NXWZ2310 genetic variation analysis

**FIGURE 2:**
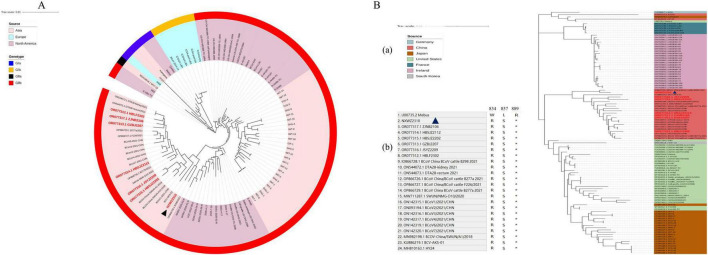
Phylogenetic analysis of BCoV NXWZ2310 strain and ORF1a. **(A)** Phylogenetic analysis of BCoV NXWZ2310 strains along with other 96 reference BCoV strains. The trees were constructed using the distance-based maximum likelihood method of the software MEGA 7.0 with 1000 bootstrap replicates, and the GenBank numbers for the reference BCoV are shown in the figure; **(B)** (a) Sequence comparison between BCoV NXWZ2310 ORF1a and the reference BCoV ORF1a, BCoV NXWZ2310 strain were aligned using Clustal W. (b) Amino acid sequence alignment of ORF1a 834, 857 and 889. Red label: The sequence was obtained in our laboratory. Black triangle: BCoV NXWZ2310 was marked with a black triangle.

**FIGURE 3:**
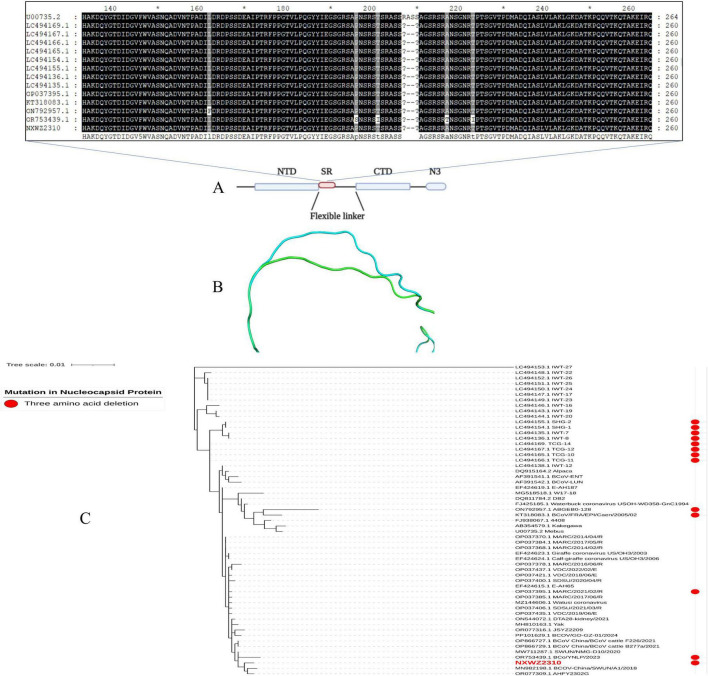
N gene mutation schematic. **(A)** Domain organization of N protein. NTD, N-terminal RNA binding domain; SR, Serine/Arginine domain; CTD, C-terminal RNA binding domain; N3, C-terminal domain (Nikolakaki and Giannakouros, 2020); **(B)** N protein homology modeling partial diagram. Mebus strain N gene sequence homology modeling diagram (blue); NXWZ2310 N gene sequence homology modeling diagram (green); **(C)** Phylogenetic analysis of the NXWZ2310 N gene, along with 56 other reference sequences, was performed using the Maximum Likelihood method.

Ninety-seven BCoV genomic sequences were used for phylogenetic analyses and indicated that BCoV NXWZ2310 strain belonged to the GIIb genotype, the dominant genotype in China. It was closest to B298/2021 (GenBank: OP866728.1) on the phylogenetic tree (Figure 2A), reaching 99.06% nucleotide similarity. The ORF1 gene had three unique amino acid mutations at sites 834, 857 and 889 (Figure 2B). Additionally, the N gene contained three-amino-acid deletions in the regions rich in arginine and serine (Figure 3A), which led to truncation of the N protein flexible junction site (Figure 3B). The mutations in the N gene detected in different countries cluster together in different branches (Figure 3C).

### 3.3 Clinical symptoms of calves and viral shedding

**FIGURE 4:**
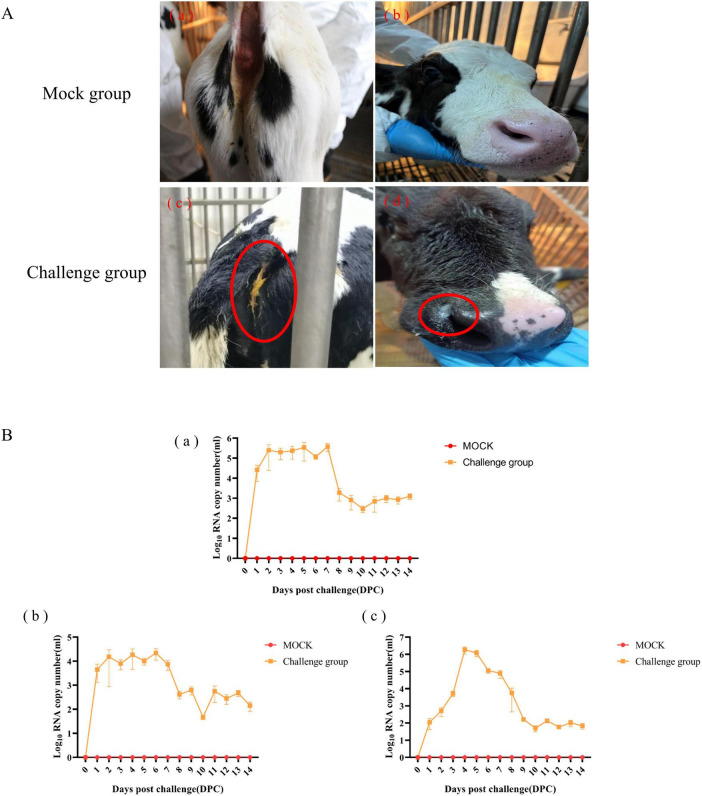
Clinical Symptoms and viral shedding of 7-day-old calves challenged with BCoV NXWZ2310 strain. **(A)** (a, b) Asymptomatic healthy cattle from mock group; **(A)** (c) Watery diarrheal; **(A)** (d) Nasal discharge. **(B)** (a) Nasal swab; (b) Throat swab; (c) Rectal swab. Clinical symptoms have been circled by red oval.

The pathogenicity of BCoV NXWZ2310 strain was assessed in 7-day-old calves. Prior to the challenge, the calves were healthy and exhibited no clinical symptoms. However, at 4 DPI, the challenge group began to show nasal discharge and diarrhea. After 4 DPI, five calves developed watery diarrhea, and four sequentially developed nasal discharge, followed by a gradual subsidence of these symptoms (Figure 4A; Table 2).

**TABLE 2:** Clinical symptoms observed in 7-days-old calves.

Day post challenge	Challenge group						Mock group					
	1	2	3	4	5	6	1	2	3	4	5	6
1	–	–	–	–	–	–	–	–	–	–	–	–
2	D	–	–	–	–	–	–	–	–	–	–	–
3	–	–	–	–	–	D	–	–	–	–	–	–
4	D	D	D	–	–	C	–	–	–	–	–	–
5	D, C	D	D	D	D	D, C	–	–	–	–	–	–
6	D, N	D, N	D	–	–	D, C	–	–	–	–	–	–
7	D	N	D	D	D	D, C	–	–	–	–	–	–
8				D	D	D, C				–	–	–
9				N	N	–C				–	–	–
10				D, N	–	–				–	–	–
11				D, N	D	D				–	–	–
12				–	–	–				–	–	–
13				–	–	D				–	–	–
14				–	D	D				–	–	–

D, Watery diarrhea; N, Nasal discharge; C, Cough.

Viral shedding was determined from 1 to 14 DPI; it peaked at 10^6.228^ copies/mL from the nasal swabs at 7 DPI, 10^5.0^ copies/mL from the throat swabs at 6 DPI, and 10^6.692^ copies/mL from the rectal swabs at 4 DPI. Subsequently, lower levels of viral shedding were detected up to 14 DPI. No BCoV copies were detected in the control group throughout the experiment (Figure 4B). These results indicated that BCoV NXWZ2310 strain caused respiratory and digestive tract symptoms.

### 3.4 Gross lesions and viral distribution

**FIGURE 5:**
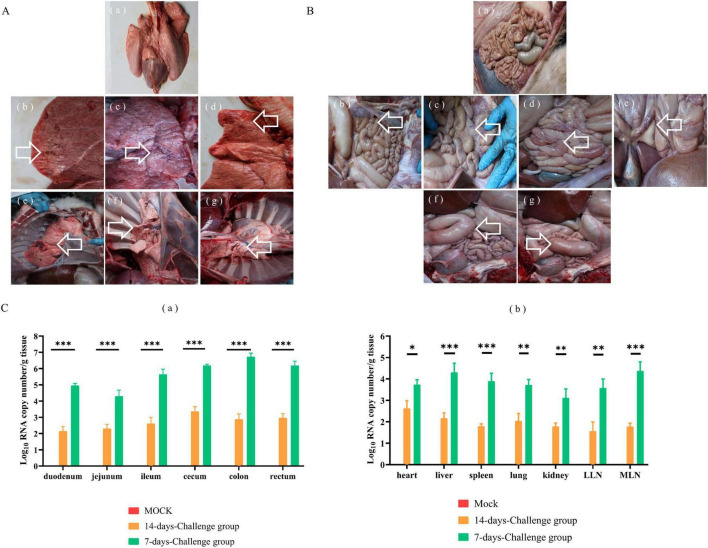
Gross examination and virus distribution analysis from 7-day-old calves challenged with NXWZ2310 isolate. The autopsy of the lung lesion was labeled with white arrows. **(A)** (a) Lung of mock group; (b–g) Partial parenchymal lesions of pulmonary lobe of challenge group. **(B)** (a) Intestines of control group; (b) Intestinal wall thinning; (c) The enlargement of lymph node; (d) Congestion of jejunum and flatulence of colon; (e) Congestion of duodenum; (f) Flatulence of duodenum; (g) Flatulence of cecum. **(C)** (a) Viral load in intestines; (b) Viral load in heart, liver, spleen, lungs, kidneys, hilar lymph nodes, mesenteric lymph nodes. Data presented as means ± SD; a notable difference exists in the mean values denoted by distinct maker: *P < 0.05; **P < 0.01, ***P < 0.0001.

Necropsy examinations at 7 DPI in the challenge group revealed partial parenchymal lesions of the pulmonary lobe (Figure 5A), thinning of the intestinal wall (Figure 5B), enlargement of mesenteric lymph nodes (Figure 5B), congestion of jejunum, colonic flatulence (Figure 5B), and congestion of duodenum (Figure 5B). Intestinal flatulence occurred at 14 DPI in the challenge group (Figure 5B). The ileum, cecum, colon, and rectum exhibited higher viral copy loads, followed by varying degrees of detection in other tissues. At 7 DPI, the intestinal viral copy loads were 10^4.942 ± 0.082^ copies/g for the duodenum, 10^3.545 ± 0.786^ copies/g for the jejunum, 10^5.400 ± 0.343^ copies/g for the ileum, 10^6.183 ± 0.0535^ copies/g for the cecum, 10^6.602 ± 0.269^ copies/g for the colon, and 10^6.074 ± 0.219^ copies/g for the rectum. The intestinal viral copy loads were lower at 14 DPI than at 7 DPI, at 10^2.020 ± 0.225^ copies/g for the duodenum, 10^2.150 ± 0.284^ copies/g for the jejunum, 10^2.151 ± 0.452^ copies/g for the ileum, 10^2.942 ± 0.577^ copies/g for the cecum, 10^2.684 ± 0.284^ copies/g for the colon, and 10^2.880 ± 0.191^ copies/g for the rectum (Figure 5C). For the lungs, the viral copy load was 10^3.630 ± 0.187^ copies/g at 7 DPI, then 10^1.807 ± 0.389^ copies/g at 14 DPI. The viral copy loads in the heart, liver, spleen, kidneys, mesenteric lymph nodes and lung lymph nodes were significantly higher at 7 DPI than at 14 DPI (Figure 5C).

### 3.5 Histopathological lesions in the lungs and intestines

**FIGURE 6:**
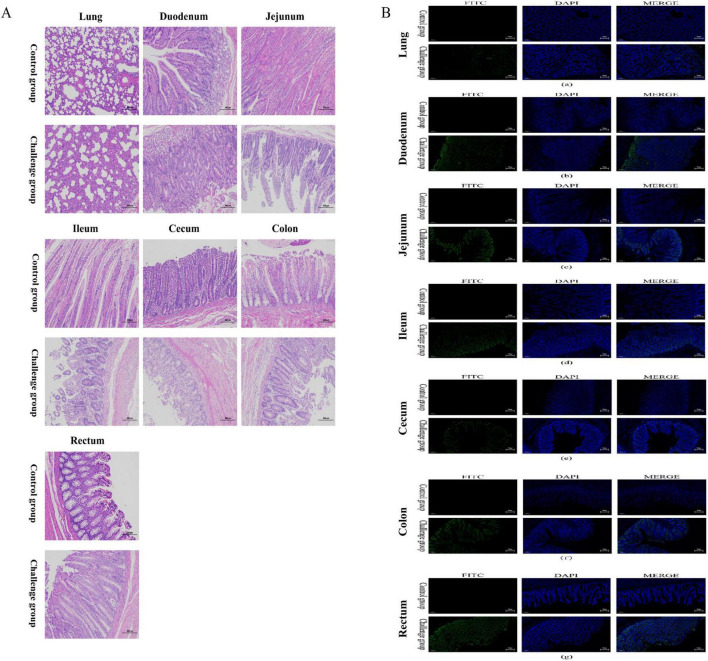
Histopathological lesion and immunofluorescence analysis of intestinal tissues and lung from 7-day-old calves challenged with BCoV NXWZ2310 strain. **(A)** Representative of the hematoxylin and eosin-stained lung, duodenum, jejunum, ileum, cecum, colon and rectum tissue sections of inoculated calves. **(B)**: immunofluorescence analysis of intestinal tissues and lung.(a) Lung; (b) Duodenum; (c) Jejunum; (d) ileum; (e) cecum; (f) colon; (g) rectum. Scale bar, 200 μm.

Microscopic lesions in the lungs and intestines were also analyzed. Compared with the calves in the mock group, those in the challenge group exhibited intestinal villous atrophy, sloughed intestinal villi, eosinophilia, and widened interstitial space in the lungs (Figure 6A).

IFA detection of BCoV confirmed the presence of the virus in the cytoplasm of epithelial cells on the atrophied villi and sloughed villi in the intestinal segments, as well as in the lung interstitium in the challenge group (Figure 6B). These results further demonstrate that BCoV NXWZ2310 strain is pathogenic in the intestines and lungs.

## 4 Discussion

BCoV was first reported in 1984 and is now widespread across the world (Zhu et al., 2022b). But it is difficulty to isolated BCoV from positive samples, which may be due to the presence of noninfectious virus particles or low viral load in these samples. Besides, the amount of trypsin in HRT-18 cells might also contribute to successful BCoV isolation. In the present study, fecal samples were confirmed as BCoV-positive, and used to isolate virus in HRT-18 cells. Then, BCoV NXWZ2310 strain was successfully isolated. After several passages, the viral titer reached at 10^6.0 ± 0.191^ TCID_50_/mL at P6, showing that the BCoV NXWZ2310 strain was highly replicative in HRT-18 cells.

To characterize the virus isolate, the complete genome of BCoV NXWZ2310 strain was sequenced and analyzed. Based on the phylogenetic tree analysis, BCoV NXWZ2310 strain can be clustered into one clade with other isolates from China, indicating that BCoV strains currently circulating in multiple Chinese provinces are closely related. Recombination and mutation are regarded as critical mechanisms in viral evolution, particularly for coronaviruses, driving alterations in pathogenicity, host range, and transmission routes. It has posed a significant threat to both human and animal life. In this study, BCoV NXWZ2310 strain exhibited three-amino-acid deletions on the N gene, which likely contributed to its pathogenicity, allowed it to adapt to different hosts, and caused viral recombination. Deletions on the BCoV-N gene have been detected in Japan (GIIb lineages), France (GIb lineages), the United States (GIIb lineages) and China (GIIb lineages) (GenBank numbers: LC494169, LC494167, LC494166, LC494165, LC494155, LC494154, LC494136, LC494135, OP037395, KT318083, OR753439, and ON792957). However, phylogenetic tree analysis showed that they do not form smaller clustered branches like those seen with the HE gene deletion (Workman et al., 2022). Thus, convergent evolution may explain the 3-amino-acid deletion in the different BCoV lineages, and similar structures can develop in unrelated lineages. Moreover, prosperous cattle trade and cattle-related products between countries may play a crucial role in the mutation and spread of BCoV. Further studies are needed to trace the origins of the NXWZ2310 strain and to better understand the transmission dynamics of BCoV. There is an urgent need to uncover the pathogenicity of the N gene deletion strain.

On the other hand, structural analysis showed that the BCoV NXWZ2310 strain isolated in this study is a mutant of the N gene, with deletions occurring in the regions rich in arginine and serine (RS/SR; Figure 3). The RS/SR domain also plays an important role in the occurrence and development of various diseases. For example, abnormal splicing events are caused by dysfunction of SR proteins and are associated with the occurrence of many diseases, such as cancer and neurodegenerative diseases (Prescott et al., 2014). Whether the deletion of an arginine and serine in the RS/SR domain of the BCoV-N protein affects the function of the RS/SR domain and immune response requires further investigation.

The identification of BCoV as a respiratory pathogen is controversial because healthy cattle carry BCoV. However, the causes of respiratory symptoms due to BCoV have been reported previously. In calves infected by the virulent BCoV strain, viral shedding peaked with 10^6.6^ copies/mL in nasal swabs, and histopathologic lesions in the upper and lower respiratory tissues were detected, revealing that BCoV is a respiratory pathogen (Soules et al., 2022). Moreover, a Korean WD-BCoV strain was used to infect colostrum-deprived calves, and epithelial damage was observed, and viral antigens were detected in their digestive and respiratory tracts (Soules et al., 2022; Park et al., 2007). In this study, we isolated BCoV NXWZ2310 from feces and systemically evaluated its pathogenicity in the respiratory and digestive tracts. Our results showed that BCoV NXWZ2310 strain can cause severe respiratory and digestive disease in calves. High levels of BCoV copies were detected in nasal, throat, and rectal swabs, and varying loads of BCoV were detected in all intestinal lesions and other tissues and organs such as the heart, liver, spleen, lungs, kidneys, lung lymph nodes, and mesenteric lymph nodes. These results were consistent with previous findings and confirmed that BCoV NXWZ2310 strain has dual tropism and induces pathological changes in the digestive and respiratory tracts of calves. Notably, BCoV NXWZ2310 strain induced small and large intestinal lesions differed from those caused by PEDV and TGEV, which mainly cause small intestinal lesions (Kim and Chae, 2003; Kim and Chae, 2002). Furthermore, no BCoV NXWZ2310 RNA was detected in the serum, and rectal temperatures remained normal in the infected calves throughout the study. However, due to the absence of comparative experiments between the wild-type strain and the strain with the N gene deletion, we are unable to draw a definitive conclusion regarding whether the deletion in the N gene is associated with pathogenesis and virulence.

In conclusion, we successfully isolated BCoV NXWZ2310 with a deletion on the N gene and serially passaged it in HRT-18 cells. Some of its biological characteristics and pathological capabilities were investigated for the first time. This strain caused severe respiratory and digestive disease of calves. These results provide a foundation for further understanding the pathogenicity of the emerging BCoV with N gene deletion.

## Data Availability

The datasets presented in this study can be found in https://www.ncbi.nlm.nih.gov. The accession numbers can be found in the article/supplementary material.
